# Genetic Structure of Chimpanzee Populations

**DOI:** 10.1371/journal.pgen.0030066

**Published:** 2007-04-20

**Authors:** Celine Becquet, Nick Patterson, Anne C Stone, Molly Przeworski, David Reich

**Affiliations:** 1 Department of Human Genetics, University of Chicago, Chicago, Illinois, United States of America; 2 Broad Institute of Harvard and MIT, Cambridge, Massachusetts, United States of America; 3 School of Human Evolution and Social Change, Arizona State University, Tempe, Arizona, United States of America; 4 Department of Genetics, Harvard Medical School, Boston, Massachusetts, United States of America; University of Oxford, United Kingdom

## Abstract

Little is known about the history and population structure of our closest living relatives, the chimpanzees, in part because of an extremely poor fossil record. To address this, we report the largest genetic study of the chimpanzees to date, examining 310 microsatellites in 84 common chimpanzees and bonobos. We infer three common chimpanzee populations, which correspond to the previously defined labels of “western,” “central,” and “eastern,” and find little evidence of gene flow between them. There is tentative evidence for structure within western chimpanzees, but we do not detect distinct additional populations. The data also provide historical insights, demonstrating that the western chimpanzee population diverged first, and that the eastern and central populations are more closely related in time.

## Author Summary

Common chimpanzees have been traditionally classified into three populations: western, central, and eastern. While the morphological or behavioral differences are very small, genetic studies of mitochondrial DNA and the Y chromosome have supported the geography-based designations. To obtain a crisp picture of chimpanzee population structure, we gather far more data than previously available: 310 microsatellite markers genotyped in 78 common chimpanzees and six bonobos, allowing a high resolution genetic analysis of chimpanzee population structure analogous to recent studies that have elucidated human structure. We show that the traditional chimpanzee population designations—western, central, and eastern—accurately label groups of individuals that can be defined from the genetic data without any prior knowledge about where the samples were collected. The populations appear to be discontinuous, and we find little evidence for gradients of variation reflecting hybridization among chimpanzee populations. Regarding chimpanzee history, we demonstrate that central and eastern chimpanzees are more closely related to each other in time than either is to western chimpanzees.

## Introduction

Standard taxonomies recognize two species of chimpanzees: bonobos *(Pan paniscus)* and common chimpanzees *(P. troglodytes),* whose current ranges in Africa do not overlap. Common chimpanzees have been classified further into three populations or subspecies based on their separation by geographic barriers (generally rivers): western *(P. troglodytes verus),* central *(P. t. troglodytes),* and eastern *(P. t. schweinfurthii)* [1,2]. While there are no or only slight morphological or behavioral differences among the common chimpanzees [3–5], genetic studies of mitochondrial DNA (mtDNA) [6,7] and the Y chromosome [8] have supported the geography-based population designations [6,8], and mtDNA studies have led to the proposal of a fourth common chimpanzee subspecies, *P. t. vellorosus*, around the Sanaga river in Cameroon [9,10]. However, studies of single loci provide at best partial information about history and population subdivision [11]; for example, analyses of X and Y chromosome datasets [12] suggest that genetic diversity is highest in central and lowest in western chimpanzees, while mtDNA suggests a different pattern [8]. Resequencing and microsatellite-based datasets have also provided inconsistent evidence about whether eastern chimpanzees are more diverse than bonobos [5,14]. To obtain a clear picture of chimpanzee population structure, a large number of independently evolving regions should be studied simultaneously.

The most comprehensive study of chimpanzees to date—including multiple loci and samples from western, central, and eastern chimpanzees and bonobos—found few fixed genetic differences among chimpanzee populations and estimated autosomal *F*_ST_ values between populations of 0.09–0.32, overlapping the range of differentiation seen in humans. Fischer et al. argued from these results that there are no chimpanzee subspecies and suggested instead that chimpanzee variation might be characterized by continuous gradients of gene frequencies, with ongoing gene flow across groups [5]. This and the other multilocus datasets that have been published to date [13–15] are small compared with recent genetic assessments of human structure [16], however, and have not yet provided a clear picture. For example, mtDNA and Y chromosome data have been interpreted as showing discontinuity among chimpanzee populations [6–10], potentially at odds with the model proposed by Fischer et al. [5].

An accurate picture of chimpanzee population structure is also crucial for understanding their history. For example, Won and Hey estimated that common chimpanzees and bonobos split ∼0.9 million years ago (Mya), and western and central chimpanzees split ∼0.42 Mya, with low levels of migration from western to central since that time [17]. This analysis, which assumed that the populations split from a common ancestor, would need to be reevaluated if the data were better described by a model of stable isolation by distance [5].

To clarify chimpanzee population structure, we gathered an order-of-magnitude larger dataset than has previously been available. This allowed us to test whether genetic data alone can be used to assign chimpanzees to the categories of western, central, and eastern chimpanzees, whether there is evidence for substantial admixture between groups, and whether there is unrecognized substructure among the chimpanzees [18].

## Results/Discussion

We analyzed data from 310 polymorphic microsatellites in 84 individuals: 78 common chimpanzees and six bonobos. These samples were chosen to include multiple representatives of each putative population. Of the common chimpanzees, 41 were reported as western, 16 as central, seven as eastern, three as hybrids, and 11 did not have a reported subpopulation (Table 1). This dataset was designed to include a similar number of genetic markers (and in fact included many of the same markers) as the dataset analyzed by Rosenberg et al. to elucidate human population structure [16]. Because of high mutation rates, microsatellite alleles often have arisen multiple times, and hence it is difficult to resolve the genealogy at any locus. A benefit of the high mutation rate, however, is that microsatellites provide more information about recent historical events per locus compared with resequencing data [19].

**Table 1:**
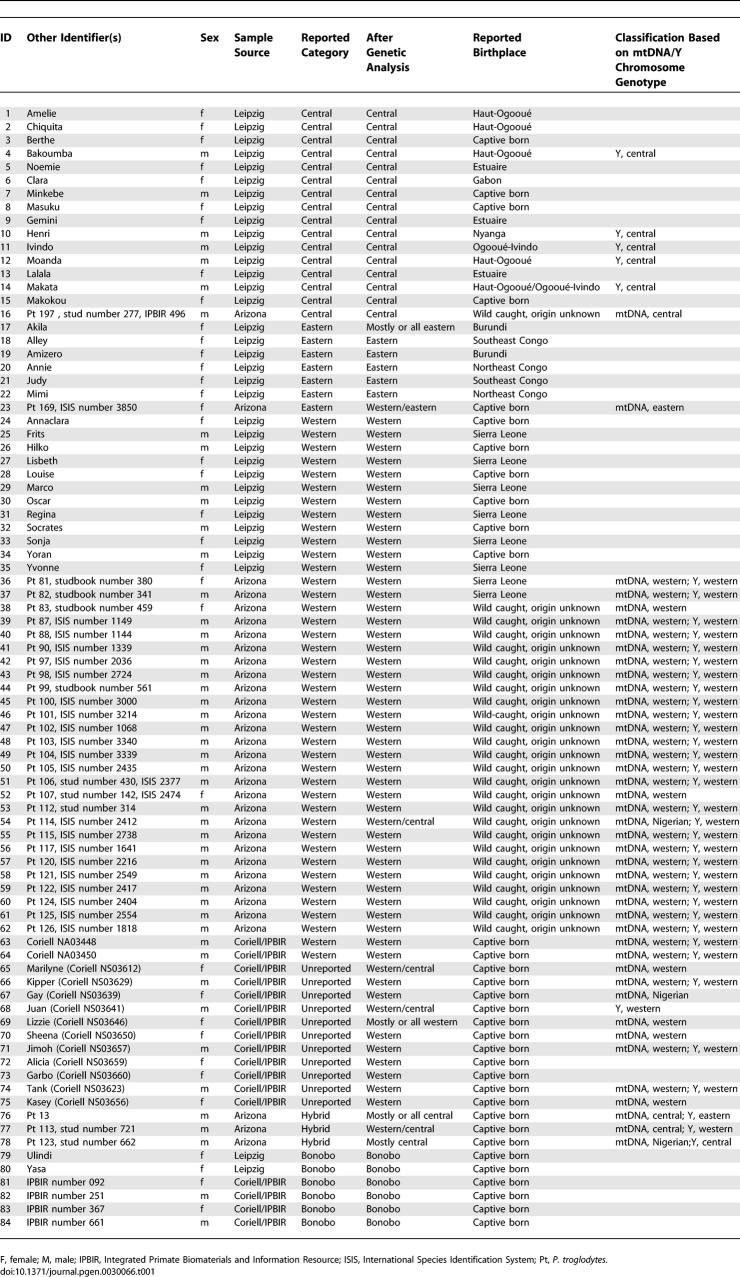
Details of the 84 Samples in This Study

### Cluster Analysis

**Figure 1:**
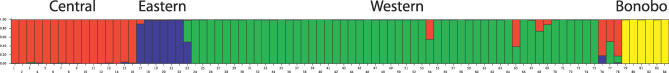
STRUCTURE Analysis, Blinded to Population Labels, Recapitulates the Reported Population Structure of the Chimpanzees Individuals 76–78 are reported hybrids. Only two individuals with a >5% proportion of ancestry in more than one inferred cluster are wild born: number 54 and number 17. Red, central; blue, eastern; green, western; yellow, bonobo.

To explore the genetic evidence for subdivision among chimpanzees, we first applied the program STRUCTURE to the dataset (Materials and Methods) [20]. Each STRUCTURE analysis requires a hypothesized number of populations and assigns individuals to these populations—without using any pre-assigned population labels—in a way that minimizes the amount of Hardy-Weinberg disequilibrium and linkage disequilibrium across widely separated markers. The analysis strongly supports the division of the samples of common chimpanzees and bonobos into at least four discontinuous subpopulations. Although the software does not provide a formal statistical procedure for choosing the number of clusters, Pritchard et al. [20] suggest using the model with the highest likelihood. When we ran the software assuming models of one to six clusters (averaging results for three random number seeds for each model), the likelihood of the data for four clusters was higher than for any other model. The inferred clusters correspond remarkably well to the reported labels of western, central, eastern, and bonobo, and also agree well with the assignments based on mtDNA or Y chromosome haplotypes (Figure 1; Table 1).

The multilocus dataset also provides power to identify individuals with multiple ancestries and to assess their ancestry proportions. This cannot be done reliably using studies of single loci such as the Y chromosome or mtDNA, because individuals can in fact be descendants of multiple ancestral populations without carrying DNA from some of the populations at the locus being studied. The STRUCTURE analysis identified nine individuals as having more than 5% genetic ancestry from two clusters (Table 2).

**Table 2:**
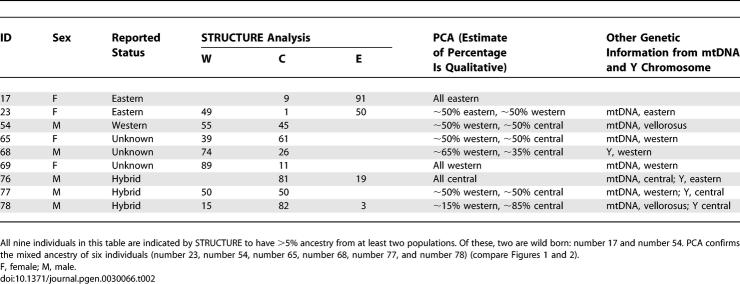
Individuals with >5% Ancestry from More than One Cluster

**Figure 2:**
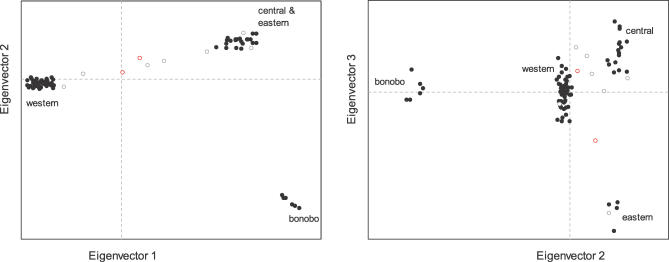
PCA, Without Using Population Labels, Divides the 84 Chimpanzees into Four Apparently Discontinuous Populations of Western, Central, Eastern, and Bonobo Plots of eigenvectors 1 versus 2, and eigenvectors 2 versus 3, show clustering into populations, with the expected assignments for the 75 individuals identified as all of one ancestry by STRUCTURE (solid circles). The nine individuals identified by STRUCTURE as hybrids (open circles) are for the most part identified as hybrids by PCA as well. There are two individuals (red open circles) reported as being of a particular population but that in fact appear to be hybrids: number 23, reported as eastern but in fact a western-eastern hybrid, and number 54, a wild-born individual reported as western but in fact a western-central hybrid.

Of the individuals identified by STRUCTURE as likely hybrids, seven were born in captivity, and just two were wild-caught, consistent with what would be expected if there were low rates of migration between central and western chimpanzees in the wild (Table 2) [17]. Interestingly, individual number 54, one of two wild-caught individuals identified as a hybrid by this analysis, has an mtDNA haplotype hypothesized to correspond to *P. t. vellorosus* [9]. The two captive-born chimpanzees with the putative *P. t. vellorosus* haplotype, however, have markedly different estimates of ancestry proportions, and thus there is no evidence from the STRUCTURE analysis that these individuals form a distinct population: the population ancestry estimates are 45% central and 55% western for number 54; 84% central and 16% western for number 78; and 100% western for number 67.

We also used STRUCTURE to validate a minimal set of markers that could be useful for classifying chimpanzees in conservation studies (Table S1). The top 30 markers (ranked by informativeness for assigning individuals to populations [19]) provide excellent power for classification. Of 75 chimpanzees estimated as having 100% ancestry in one group by all markers, we found that 71 were classified identically by the top 30 markers (by the criterion that at least 90% of the ancestry is assigned to the same group). Of nine individuals identified as hybrids with all the markers, six were also detected as hybrids with the reduced set. In addition to quantitative precision, the microsatellite panel also has a qualitative advantage over single marker studies in classifying chimpanzee hybrids: mtDNA and Y chromosome analyses cannot detect first generation female hybrids (Table 1) or reliably classify hybrids of the second or higher generation.

### Principal Components Analysis

We next carried out principal components analysis (PCA). This approach has been shown to have similar power to capture population structure as STRUCTURE, but also provides a formal way of assigning statistical significance to population subdivision [21]. When the PCA is applied to the chimpanzee data, the results support four discontinuous populations into which almost all chimpanzees and bonobos can be classified. The first three principal components (eigenvectors) are all highly statistically significant (*p* < 10^−12^) and nearly perfectly separate western, central, and eastern chimpanzees, and bonobos (Figure 2). Only six chimpanzees fall visually outside of the clusters, a subset of the nine identified by STRUCTURE as having at least 5% genetic contribution from more than one population (Table 2). The fourth eigenvector (*p* = 0.011) is also significant, and the fifth is not significant (*p* = 0.44).

The eigenvectors are strongly correlated to the population labels. We used nonparametric analysis (Kruskal-Wallis tests) to explore whether the values of each sample along the four significant eigenvectors were significantly correlated to the four pre-existing population labels. The overall statistic is highly significant (*p* < 10^−10^) for the first three eigenvectors but insignificant for the fourth (*p* = 0.97), indicating that this eigenvector is capturing population subdivision that is different from the traditional western/central/eastern/bonobo designations.

To explore whether the fourth eigenvector might reflect an as-yet-undefined chimpanzee population, we carried out analyses separately on the western chimpanzee (*n* = 49), central chimpanzee (*n* = 16), eastern chimpanzee (*n* = 6), and bonobo (*n* = 6) samples (including all individuals that were clearly classified by both PCA and STRUCTURE). Western chimpanzees are the only population with evidence for internal substructure (*p* = 5.5 × 10^−5^). The first eigenvector obtained when western chimpanzees are analyzed by themselves strongly correlates to the fourth eigenvector in the main analysis (*r* ^2^ = 0.92; *p* < 10^−12^) (Figure S1), indicating that the fourth eigenvector describes subdivision within western chimpanzees.

Although the fourth eigenvector seems to be detecting real structure, it does not mark out discontinuous subpopulations of western chimpanzees (Figure S1). The failure to reveal the details of the structure is evident not only in the PCA, but also in an application of STRUCTURE to the western chimpanzees only, in which a model of only one cluster is most likely. There is also no pattern to the classification of western chimpanzees even when we consider a model of two clusters (unpublished data). The most likely explanation is that there is not enough data to assign individuals to different ancestries. Understanding of the fourth eigenvector in the PCA will require more genetic data and better information about geographic origin. In particular, we note that the only wild caught western samples for which we had geographic information are from one location (Sierra Leone), thus we could not perform a test for correlation with geography.

### Testing for Additional Populations

Could there be additional population structure [18] among the chimpanzees that we have not yet detected? A particular concern is that our sample size is limited, decreasing our power to detect further structure especially among nonwestern chimpanzees.

To place an upper bound on further structure especially among the central chimpanzees, we considered the possibility that, among the 16 central chimpanzees, a subset is from a different population. We performed PCA on 10 central/6 eastern, 11 central/5 eastern, 12 central/4 eastern, 13 central/3 eastern and 14 central/2 eastern chimpanzees and assessed what fraction of 1,000 random resamplings of central and eastern chimpanzees showed evidence of structure (*p* < 0.05). This allowed us to assess power to detect an additional population as diverged as the eastern chimpanzees.

The resampling analysis found that 6, 5, 4, 3, and 2 eastern chimpanzees could be detected from amidst the central chimpanzees with 100%, 100%, 99%, 54%, and 7% probability, respectively. Since the *F*_ST_ between central and eastern chimpanzees is 0.05–0.09 (Table 2) [5], this allowed us to place an upper bound on the undetected structure that might exist among central chimpanzees given that we did not detect further structure. If the three samples with the *P. t. vellorosus* mtDNA haplotype in our study constitute members of a distinct population, their differentiation from central chimpanzees is likely to be *F*_ST_ ≤ 0.09, lower than those observed between some pairs of human populations [22]. An important caveat is that we have no power to detect population structure for chimpanzees missed by our sampling (we also have little power if there are fewer than three individuals from a population). Thus, a more geographically systematic survey, including more animals from a denser grid in Africa, may detect further structure.

### Evidence for Inbreeding

To test for inbreeding among the chimpanzees, we examined whether heterozygosity within individuals was significantly lower than would be expected from random mating in the population (Materials and Methods). Western and central chimpanzees both show evidence for a reduced number of heterozygous genotypes (*p* < 0.05) (Protocol S1) (we had too few eastern and bonobo samples to perform an informative test). A caveat is that misscoring of heterozygous genotypes, or the presence of polymorphisms under the primers used for genotyping, could both result in an artifactual excess of homozygotes. To follow up this initial evidence of inbreeding among chimpanzees, further analyses could search for multimegabase contiguous stretches of homozygosity [23].

### First and Second Generation Hybrids

To test for first and second generation hybrids, we calculated the likelihood of the data under the hypothesis that an individual is an *F*_1_ hybrid, compared with the alternative hypothesis of an older 50%–50% mixture of the ancestral populations. To test whether the individual is an *F*_2_/backcross—a mixture of an *F*_1_ with an unadmixed individual—we compared the likelihood of this model compared with the alternative hypothesis of an older 75%–25% mixture of the two ancestral populations (Materials and Methods).

Of the nine putative hybrids identified by STRUCTURE, the *F*_1_ hybrid test identifies captive-born individual number 23 (an approximately 50%–50% eastern-western hybrid by STRUCTURE analysis) as an *F*_1_, with a likelihood ratio (LR) of ∼24,000,000:1. The *F*_2_/backcross test identifies the captive-born individual number 68 (a 74%–26% western-central hybrid by STRUCTURE analysis) as an *F*_2_/backcross, with an LR of ∼37:1 (the evidence is weaker because the signal of an *F*_2_/backcross is more subtle). There are no other hybrids identified by either the *F*_1_ or *F*_2_/backcross test, suggesting that the other animals in Table 2 could descend from third generation or older admixture events, or be members of as-yet unidentified populations.

The *F*_1_ test produced a particularly intriguing pattern in number 54, a wild-caught individual with mtDNA that has been hypothesized to be diagnostic of *P. t. vellorosus* origin [9,10]. Individual number 54 is estimated to be a 55%–45% western-central mixture (Table 2) and shows an LR of 7:1 in favor of being an old mixture, compared with the alternative of a first generation *F*_1_ hybrid. However, a careful examination shows that the pattern of variation at number 54 fits neither the hypothesis of a first generation hybrid or an older mixture. To demonstrate this, we simulated 100 different western-central *F*_1_ hybrids and 100 older western-central mixtures by random sampling from the population allele frequencies. Simulated older mixtures always generated an LR of >100,000:1 relative to the alternative hypothesis of an *F*_1_. Simulated *F*_1_ hybrids always gave an LR < 1:2. The LR for individual number 54 of 7:1 falls outside of either expectation. This individual fits neither model, suggesting ancestry from an as-yet undetermined population.

### Allele Frequency Differentiation

To estimate the degree of allele frequency differentiation between chimpanzee groups, we computed the *R*_ST_ statistic, a microsatellite-based estimator of *F*_ST_ [24,25]. *R*_ST_ assumes the stepwise mutation model (SMM), in which the number of repeats changes by one or two or more units with an equal probability of increasing or decreasing. A goodness of fit test suggests that this simple model provides a reasonable match to our data (Figure S2). Encouragingly, the *R*_ST_ estimates of population differentiation, obtained based on assuming this model, are very similar to estimates of *F*_ST_ from a smaller multilocus dataset based on resequencing (Table 3) [5].

**Table 3:**
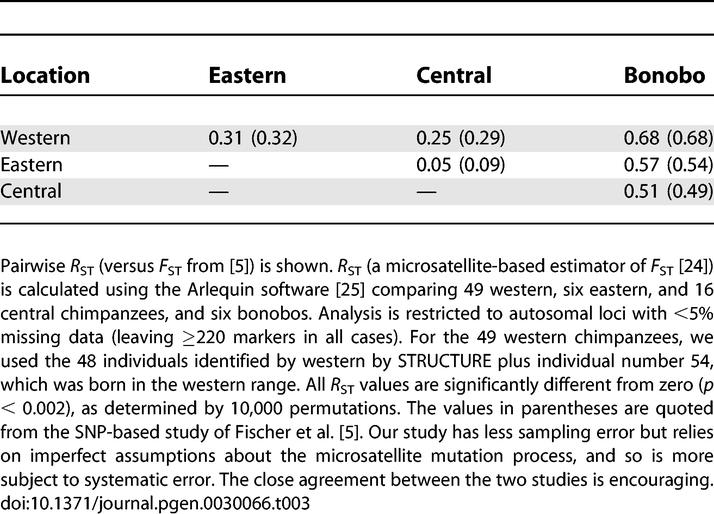
Genetic Differentiation among Populations

A particularly intriguing feature of the allele frequency differentiation results is that the allele frequency differentiation between bonobos and western chimpanzees is higher than that between bonobos and central or eastern chimpanzees (Table 3). This likely reflects greater genetic drift in the western lineage since divergence, as has also been suggested by an analysis of resequencing data [17].

### Central and Eastern Chimpanzees Are Most Closely Related in Time

The high frequency differentiation of western chimpanzees compared with other groups (Table 3) is consistent with them having been the first population to diverge, but does not prove it. An alternative explanation for the data is that there has been a smaller effective population size on the western lineage since their divergence, resulting in high genetic drift in this population. We therefore applied a formal test to assess which pair of populations is most closely related.

We approached the problem by testing whether three unrooted phylogenetic trees are consistent with the data for chimpanzees: (1) western-central and bonobo-eastern forming clades, (2) western-eastern and bonobo-central forming clades, and (3) eastern-central and bonobo-western forming clades. If a tree provides a good description of the history of the population, then the allele frequency differences between two populations should only reflect changes since they split. For example, the difference in allele frequency between central and eastern chimpanzees should have arisen entirely since their divergence from a common ancestral population and so should be uncorrelated to the allele frequency differences between western chimpanzees and bonobos.

To implement this idea, we calculated the difference in frequency within clades for all alleles and then tested for a correlation across clades. When we carry out this analysis for the first and second hypothesized trees, a correlation is observed, rejecting these trees at a significance of *p* = 0.00025 and *p* = 0.0027, respectively (Table 4). The hypothesized central-eastern/bonobo-western clade is the only one consistent with the data (*p* = 0.37). Thus, our analysis does not find any evidence for gene flow between western and central chimpanzees since their initial split, as has previously been hypothesized [17]. If gene flow did occur, it would have had to be sufficiently low to fall below the threshold of detection with our present data size and the test we applied. We conclude that eastern and central are more closely related in time than either population is to western chimpanzees.

**Table 4:**
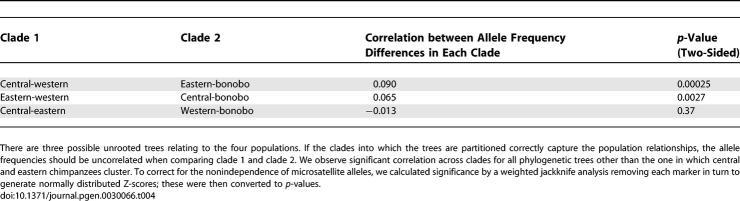
Eastern and Central Chimpanzees Phylogenetically Most Closely Related

### Population Separation Times

To estimate the times of genetic divergence among chimpanzees, we used the average squared distance (ASD) statistic [26]. For microsatellites evolving under the SMM, the expected time since the most recent common ancestor (*t*_MRCA_) of two chromosomes is expected to be ASD/2*μ,* where *μ* is the average mutation rate per year per locus, averaged across loci. Because allele lengths change according to a random walk, the ASD between allele lengths in two chromosomes is expected to increase linearly with time and is thus expected to act like heterozygosity in sequence comparisons. By averaging ASD over pairs of chromosomes within and across populations, we can estimate the average *t*_MRCA_ within and across populations.

The results confirm that genetic diversity (heterozygosity) is least for western chimpanzees and bonobos, higher for eastern chimpanzees, and highest for central chimpanzees, consistent with results obtained from a nucleotide resequencing dataset [5]. To estimate the absolute *t*_MRCA_ within and across populations, we used two estimates of the microsatellite mutation rate. The first, *μ* = 6.57 × 10^−3^ per year, relies on a 7 Mya estimated average *t*_MRCA_ between humans and chimpanzees and the observation that two western chimpanzees are ∼14.8 times less genetically diverged than humans and chimpanzees [27]. We also obtained a second estimate, 4.71 × 10^−5^ per year, based on estimated rates of microsatellite mutation in humans, and assuming 15 years per generation (Table 5).

**Table 5:**
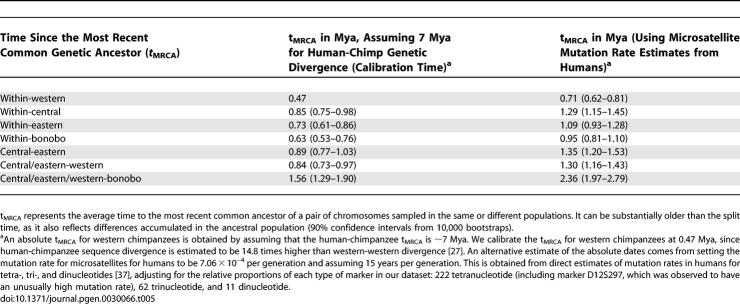
Estimates of Divergence Time from ASD

The absolute values of the time estimates for these *t*_MRCA_ *s* should be treated with caution because of uncertainty about the microsatellite mutation rate process and the calibrations used to obtain absolute dates. Nevertheless, the *t*_MRCA_ estimates are consistent with previous results from smaller resequencing based datasets [8]. We note that the central-eastern, central-western, and central-central *t*_MRCA_s are all similar, which appears at first to contradict the claim that the populations split at different times. However, most of the genetic divergence reflects ancestral diversity, which is shared among all the chimpanzees (explaining why the *t*_MRCA_ estimates are substantially older than estimates of population splitting times [14,17]). More refined analyses are needed, such as the allele frequency correlation analysis presented in Table 4, or model-based approaches, to detect the subtle patterns that indicate the splitting order of the chimpanzee populations.

### Conclusions

We have carried out the largest analysis of chimpanzee genetic variation to date, which shows that the western, central, and eastern chimpanzee subspecies designations correspond to clusters of individuals with similar allele frequencies that can be defined from the genetic data without regard to the population labels. Moreover, we find little evidence for admixture between groups in the wild. Our analysis also provides the first formal test showing that the central and eastern chimpanzee populations are more closely related to each other in time than either is to western chimpanzees.

PCA also further suggests population structure within western chimpanzees. However, more data—more samples, genetic markers, and information about geographic origin—would be needed to understand this structure. We find no support for the proposed fourth population of common chimpanzees *P. t. vellerosus*. However, the failure to detect a distinct population cluster for these individuals could simply reflect a lack of power. Our analysis does allow us to state that even if *P. t. vellorosus* is a distinct population, its level of allele differentiation from either western, central, or eastern chimpanzees is not likely to exceed *F*_ST_ = 0.09.

We finally emphasize that although we attempted to include chimpanzees from as wide a range of sites in Africa as possible, the geographic sampling of the chimpanzees that we studied was likely nonrandom. The fact that our study did not include chimpanzees from some regions—including where chimpanzees are now extinct—could create the appearance of too much discontinuity [28]. Future studies including chimpanzees across a denser grid of populations within Africa could, in principle, identify intermediate populations of chimpanzees and demonstrate more graded patterns of variation.

## Materials and Methods

### Data collection.

The samples for this study were assembled from four sources: DNA collections at the Max Planck Institute (Leipzig, Germany), Anne Stone's laboratory at Arizona State University [8], the Coriell Cell Repositories (Camden, New Jersey, United States) [29], and the Integrated Primate Biomaterial and Information Resource (Camden, New Jersey, United States) [30]. A total of 91 samples were genotyped, and 84 were included in analysis after filtering (below). We had information about the approximate geographic origin of 25 wild-caught chimpanzees (Table 1). For most remaining samples, we had a population designation, and sometimes Y chromosome and mtDNA genotypes (A. Stone, unpublished data). As far as possible, we attempted to use pedigree information to remove related individuals from the captive chimpanzees.

We assembled all the DNA samples at a single site (the Broad Institute) and carried out whole-genome DNA amplification (WGA) for all samples to generate a quantity sufficient for analysis [31]. The WGA samples were shipped to a company that specializes in genotyping microsatellite markers for human disease gene mapping studies (PreventionGenetics, http://www.preventiongenetics.com). The microsatellite markers all contain tandem repeats of two, three, or four nucleotides that vary in repeat number across individuals. For example, at a single marker, different individuals might have between three and 11 contiguous repeats of a GATA sequence. The assays used for genotyping were designed for humans. However, we hypothesized that many of them would work for chimpanzees because of the ∼98.8% sequence similarity [32].

Assays were attempted for 470 microsatellites. Most came from the Marshfield Screening Set 13 (designed for linkage screens in humans [33,34]) and were supplemented with markers from a separate mapping study. Genotyping quality was assessed by specialists at PreventionGenetics using standard measures of genotyping performance. A score of one to four was given to each marker (with one being the best and four the worst). Markers were scored as >2 because of uncertainty in allele assignment, or an excessive number of missing genotypes, or an excess in the numbers of homozygotes or noninteger alleles (defined as alleles with noninteger length differences compared with frequent alleles). We used the 310 markers that were designated as of highest or second-highest quality because the two sets produced indistinguishable inferences on our data (unpublished). For all analyses other than the use of STRUCTURE, we considered only autosomal or pseudoautosomal markers, since these could be treated uniformly. This resulted in 295 markers; we also excluded two additional pseudoautosomal markers for the PCA and *F*_1_/*F*_2_ hybrid analyses. Genotypes for all markers are available in Dataset S1.

A subset of 84 of the 91 genotyped samples were chosen for further study after removing two due to a high missing data rate, one due to evidence for contamination (more than two genotypes at many loci), and four due to evidence of genetic relatedness: two accidental duplicates, and two apparent first degree relatives. For each pair of related individuals, we dropped the one with the lower success rate. The duplicate individuals allowed us to assess genotyping quality. For the two individuals studied in duplicate, 1.18% of genotyping calls differed, suggesting an error rate per genotype of ∼0.59% (i.e., we estimate that on average approximately two loci were mistyped per individual).

### Data analysis.

We applied two complementary methods to characterize population structure in chimpanzees. First, we used the software STRUCTURE in the “admixture” mode, so that individuals were allowed to have ancestry from multiple populations. We used a model of correlated allele frequencies, a “burn-in” of 100,000 Markov Chain Monte Carlo (MCMC) iterations, and 1,000,000 follow-on MCMC iterations.

We also analyzed the data using a new implementation of PCA [21] available online in the EIGENSOFT software package [35]. Briefly, the analysis begins with a rectangular matrix, with the 84 rows corresponding to the individuals, and the columns corresponding to the alleles (the cells give the number of copies of each allele for each individual: zero, one, or two). To analyze the data, we perform a singular value decomposition, a procedure that produces eigenvectors and eigenvalues. The first eigenvector separates the samples in a way that explains the largest amount of variability, while the second and subsequent ones explain lesser amounts of variability. Thus, the first eigenvector distinguishes individuals from the population that is most differentiated, and each subsequent eigenvector separates the next most differentiated population. Eigenvalues above a significance cutoff represent significant population structure for the associated eigenvector [21].

To examine evidence for inbreeding in these samples, we used the output of STRUCTURE to assign individuals to populations, excluding individuals with >5% ancestry from more than one population and focusing our study on the population samples with more than ten individuals (treating captive and wild-caught individuals separately). Thus, we analyzed 48 western chimpanzees, including 34 wild-caught and 14 captive-born individuals. We also analyzed 13 wild-caught central chimpanzees (Table 1). We considered two statistics: *H*_w_, the average heterozygosity within an individual's two chromosomes, and *R*_w_, calculated as 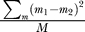 , where *m*_1_ and *m*_2_ are the alleles' number of repeat units at marker *m* within an individual, and *M* is the total number of markers considered. We used the average value of *H*_w_ and *R*_w_ over individuals in a population to test the hypothesis of random mating and assessed significance by a permutation test. Specifically, we generated 1,000 samples of *n* individuals, randomly assigning to each of them two alleles from the pool, then counted how often *H*_w_ or *R*_w_ was as small or smaller than observed. *H*_w_ was significantly lower than expected for all samples considered (at the *p* < 0.05 level), while *R*_w_ was significantly lower in wild-caught western chimpanzees (Protocol S1).

For other analyses in which we were interested in studying only individuals identified unambiguously as being from one population, we excluded the captive-born individuals defined as putative hybrids by STRUCTURE.

### Application of the stepwise mutation model.

We examined whether our data fit the SMM in the 49 individuals that are identified as western chimpanzees by both STRUCTURE and PCA. We focused on western chimpanzees because they have the largest sample size and hence provide us with the most power to detect a departure from the SMM. Under the one-step SMM, 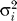 , the variance of an allele with *i* repeats, is an estimator of 2*Nμ,* the population mutation parameter [36]. For each marker, we calculated E(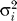 ), the expectation over all alleles of a given marker, thus obtaining an estimate of 2*Nμ* for the 221 tetra-, 62 tri-, and 11 dinucleotides included in other analyses. Averaging this estimate over all markers of the same type and dividing by *N* = 10,000 [17], we obtain an estimate of *μ* for each type of marker, 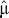 = 3.77 × 10^−3^, 1.91 × 10^−3^, and 2.17 × 10^−3^, respectively. These estimates are roughly similar to independent estimates [37] based on microsatellites in humans: 6.40 × 10^−4^, 7.10 × 10^−4^, and 1.51 × 10^−3^, respectively.

To assess the goodness of fit of the SMM, we compared the observed distribution of 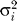 to the expected distribution, obtained by using the coalescent simulator SIMCOAL2 [38]. We generated 500 independent replicates for each type of marker under a standard neutral equilibrium model, with an effective population size [17] of 10,000, a sample size of 98 chromosomes, and a mutation rate per generation set to 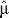 . The range constraints for the number of repeat units were set to be equal to the maximum repeat observed in the sample for each type of marker. We tested whether there was a significant difference in the distributions by an asymptotic Wilcoxon rank sum test, carrying out the test separately for each type of marker. The observed distributions do not differ significantly from the expectation under the SMM (Figure S2).

### Tests for *F*_1_ and *F*_2_/backcross hybrids.

We calculated a log Bayes-factor to test the hypothesis that a chimpanzee is an *F*_1_ hybrid of two known populations versus the alternative that it is a 50%–50% mixture (i.e., it is an older hybrid). For a given autosomal marker, one can compute a log-factor under the assumption that the allele frequencies are known; these log-factors can then be summed across all markers. In practice, our population samples are small, and so allele frequency estimates are imprecise. To account for uncertainty in the allele frequencies, we used a hierarchical Bayesian model for the unknown frequencies, with a Dirichlet prior distribution for the frequencies (the details of this calculation are similar to those described by Lockwood et al. [39]). We verified the performance of the test by simulation (see text).

## Supporting Information

Dataset S1 Raw Genotype Data in a Format Appropriate for STRUCTURE Analysis (305 KB TXT)

Figure S1 The Significant Fourth Eigenvector from the Analysis of All 84 Chimpanzees Is Correlated to the First Eigenvector from Analysis of Western Chimpanzees Only (*r* ^2^ = 0.92) Here, we present the correlation for the 49 individuals that are clearly identified as western chimpanzees by both STRUCTURE and PCA, demonstrating that these eigenvectors are revealing the same population structure.
(11 KB PDF)

Figure S2Goodness of Fit of the SMMDistributions of the expected 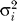 over markers for observed and simulated datasets. The distributions of E(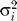 ) were computed separately for (a) 221 tetra-, (b) 62 tri-, and (c) 11 dinucleotides genotyped typed in the 49 western samples (shown in blue). In red are the results of 500 simulations for each class of microsatellites. In all three cases, the observed distribution is not significantly different from the expected distribution, as assessed by a permutation test (see Materials and Methods for more details).
(51 KB PDF)

Protocol S1 Testing for Inbreeding (51 KB PDF)

Table S1 Information on Microsatellites Used for This Study (22 KB PDF)

## Abbreviations

ASD: average squared distance
LR: likelihood ratio
mtDNA: mitochondrial DNA
Mya: million years ago
PCA: principal components analysis
SMM: stepwise mutation model
t_MRCA_: time since the most recent common ancestor
