# Supplementary material for: Genetic Structure of Chimpanzee Populations
Source: PLoS Genet. 2007 Apr 20;3(4):e66. doi: 10.1371/journal.pgen.0030066 (PMC1853122; doi:10.1371/journal.pgen.0030066)
Supplement: Supplementary file 1 [file pgen.0030066.sd002.pdf]

Protocol S1: Testing for inbreeding

Distribution of mean heterozygosity within individuals (H_w): We carry out a separate analysis for (a) 34 wild-caught, (b) 14 captive-born western chimpanzees, and (c) 13 wild-caught central chimpanzees. We assigned individuals to subpopulations according to the results of STRUCTURE, excluding individuals with >5% ancestry in more than one population. The red vertical lines are the mean H_w in each population and the blue lines are the mean H_w in permutations. The mean H_w is significantly lower (at the $P<0.05$ signififcance level) than expected by the randomized H_w in all populations (see text and table below).

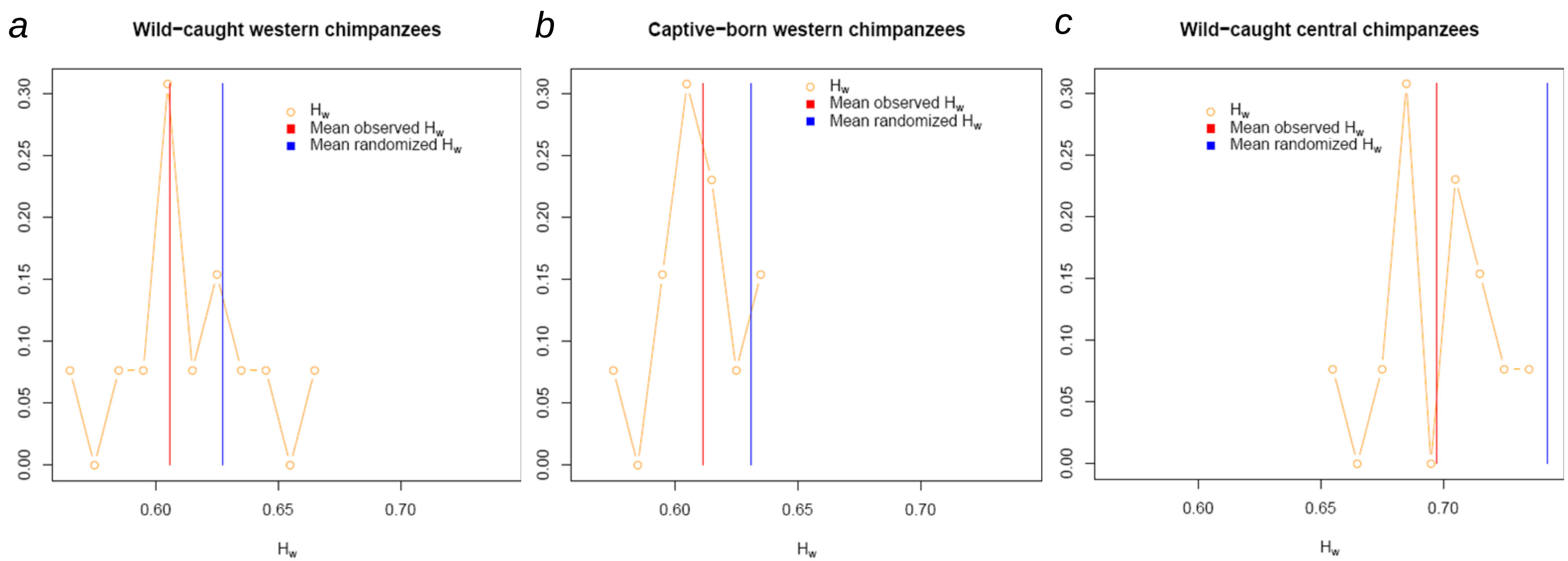

Distribution of mean squared difference in repeat units within individuals (R_w, orange): We carry out a separate analysis for (a) 34 wild-caught, (b) 14 captive-born western chimpanzees and (c) 13 wild-caught central chimpanzees. We assigned individuals to subpopulations according to the results of STRUCTURE, excluding individuals with >5% ancestry in more than one population. The red vertical lines are the mean R_w in each population and the blues lines are the mean R_w from permutation. Mean R_w is significantly lower (P<0.05) than expected by the randomized R_w in wild-caught western and central chimpanzees (see text and table below).

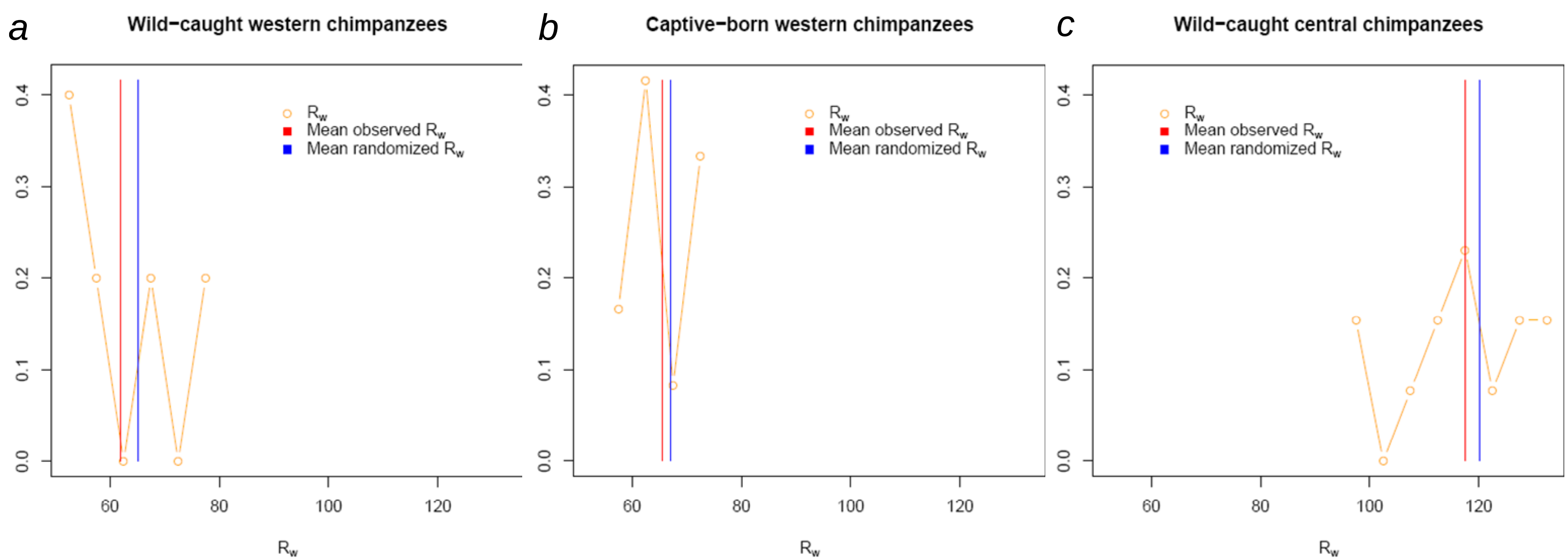

Mean heterozygosity within individuals from each population: The samples are 34 wild-caught western chimpanzees, 14 captive-born western chimpanzees and 13 wild-caught central chimpanzees. The p-value for being lower that expected, calculated from 1,000 permutations, is indicated in parentheses (see Methods).

	Wild-caught	Captive-born
Western	0.606 ($<10^{-3}$)	0.611 ($<10^{-3}$)
Central	0.697 ($<10^{-3}$)	–

Mean squared difference in repeat units within individuals in each population: The samples are 34 wild-caught western chimpanzees, 14 captive-born western chimpanzees and 13 wild-caught central chimpanzees. The p-value, calculated from 1,000 permutations, is indicated in parentheses (see Methods).

	Wild-caught	Captive-born
Western	61.9 (0.001)	65.5 (0.208)
Central	117.6 (0.186)	–
