# Supplementary material for: Genetic Structure of Chimpanzee Populations
Source: PLoS Genet. 2007 Apr 20;3(4):e66. doi: 10.1371/journal.pgen.0030066 (PMC1853122; doi:10.1371/journal.pgen.0030066)
Supplement: Supplementary file 2 [file pgen.0030066.sg001.pdf]

Supplementary Figure 1

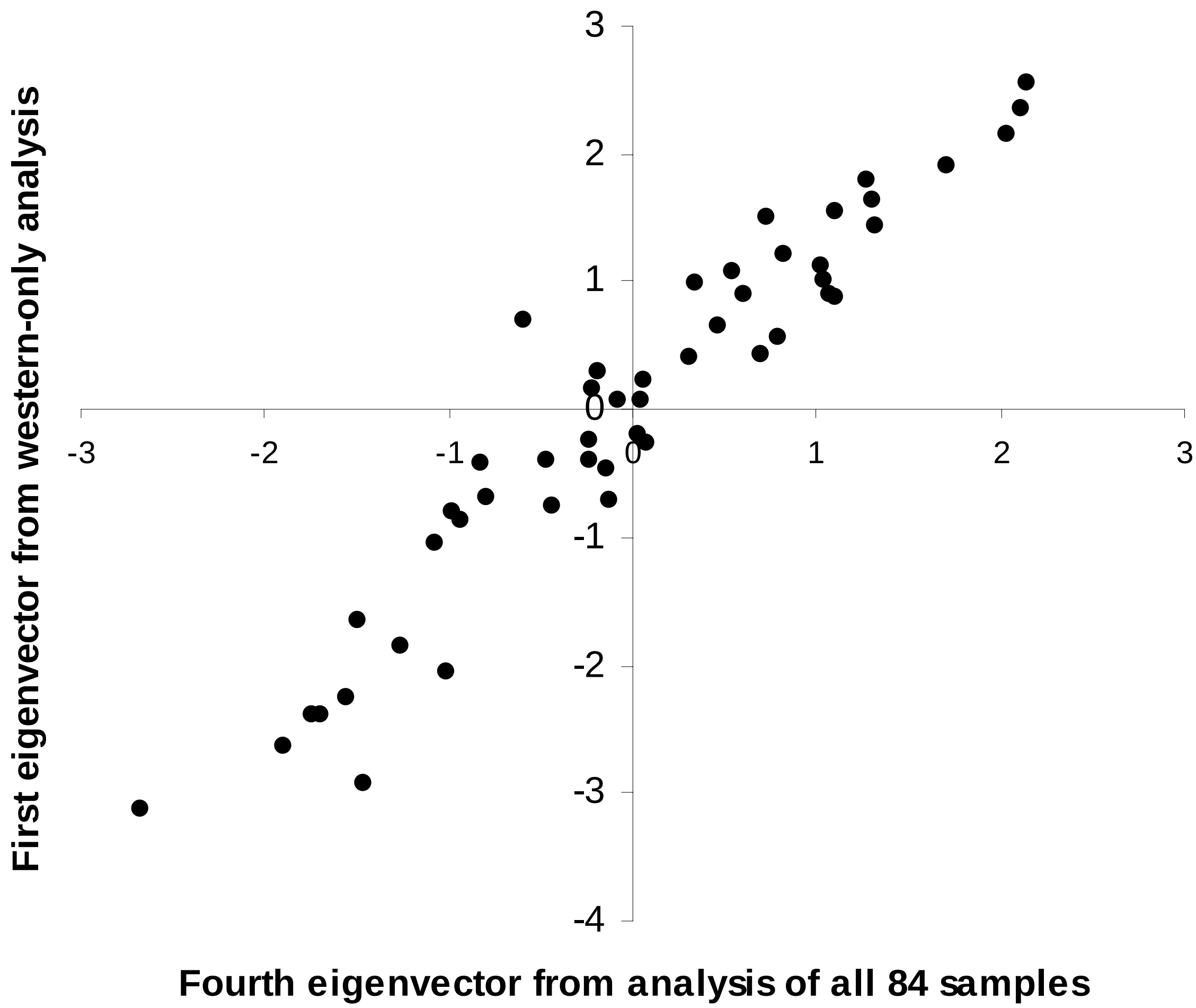

Supp. Figure 1: The significant fourth eigenvector from the analysis of all 84 chimpanzees is highly correlated to the significant eigenvector that emerges when analysis is carried out on western chimpanzees only (r^2=1). Here, we present the correlation for the 49 individuals that are clearly identified as western chimpanzees by both STRUCTURE and PCA analysis. The plot shows that these eigenvectors are clearly revealing the same population structure.
