# Supplementary material for: Genetic Structure of Chimpanzee Populations
Source: PLoS Genet. 2007 Apr 20;3(4):e66. doi: 10.1371/journal.pgen.0030066 (PMC1853122; doi:10.1371/journal.pgen.0030066)
Supplement: Supplementary file 3 [file pgen.0030066.sg002.pdf]

Supplementary figure 2:

Distributions of the expected σ_i^2 over markers, for observed and simulated data sets. The distributions of E(σ_i^2) were computed separately for 221 tetra- (a), 62 tri- (b) and 11 di-nucleotides (c) genotyped typed in the 49 western samples (shown in blue). In red are the results of 500 simulations for each class of microsatellite. In all three cases, the observed distribution is not significantly different from the expected distribution, as assessed by a permutation test (see Methods for more details).

a.

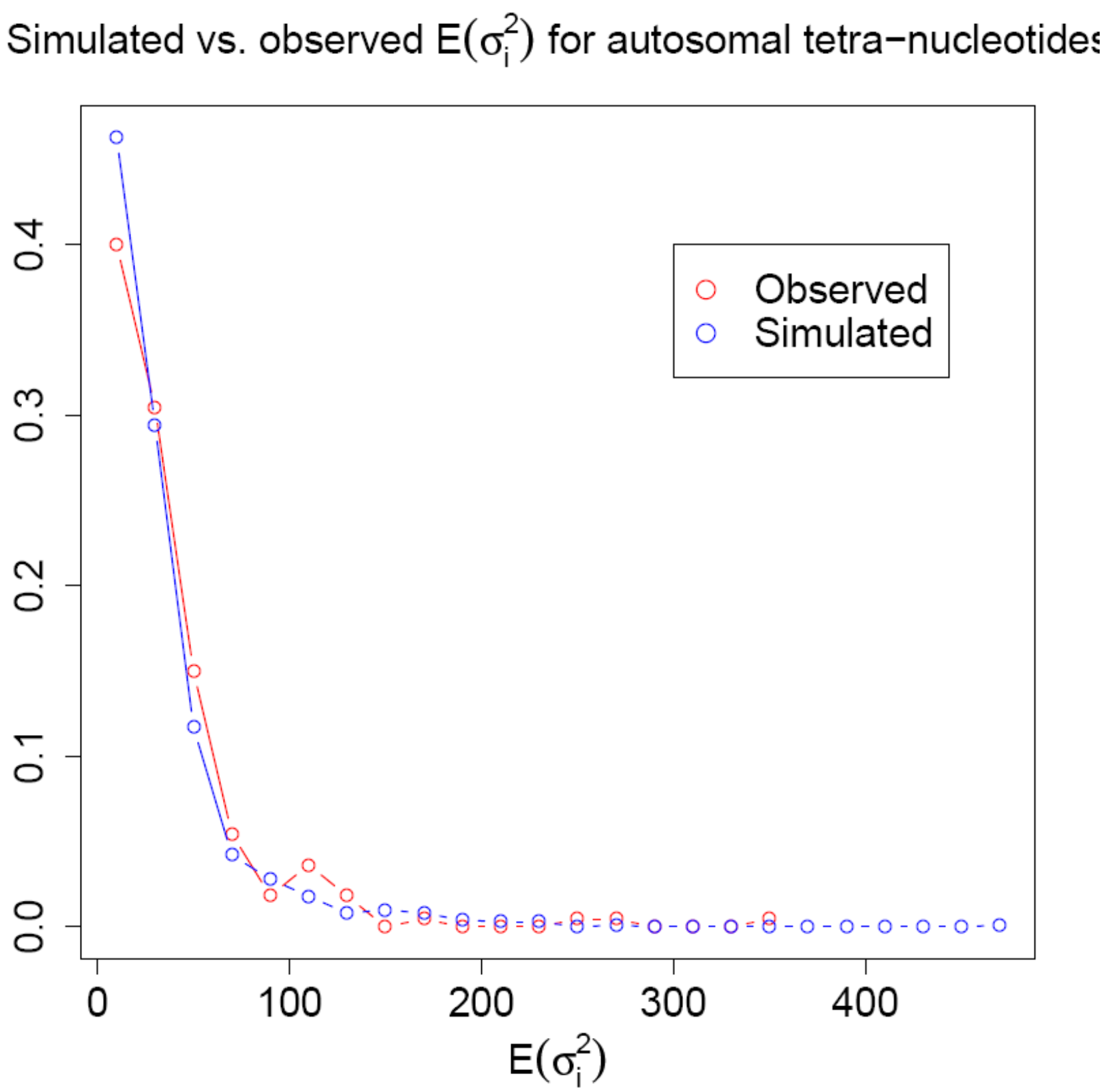

b.

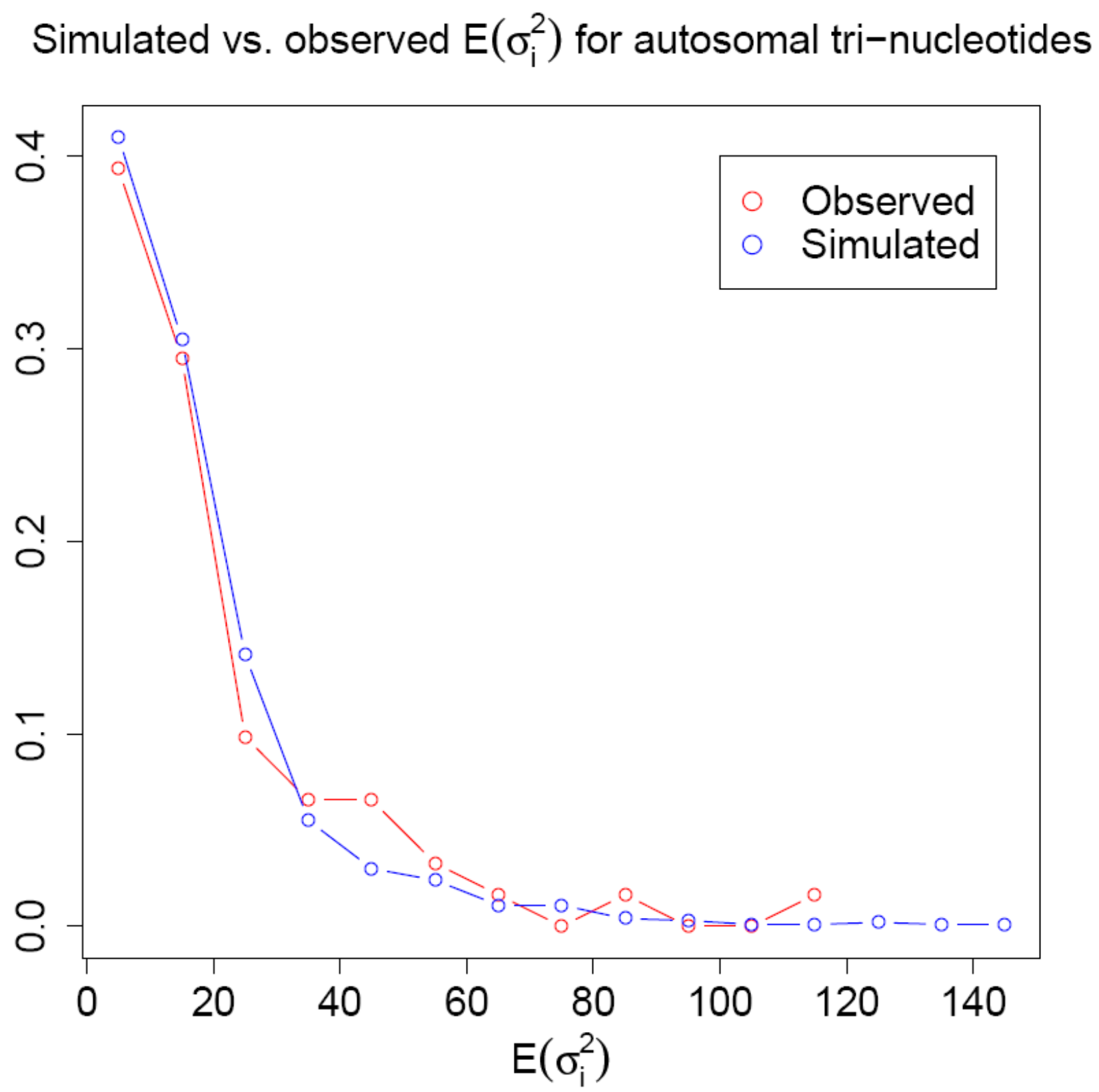

c.

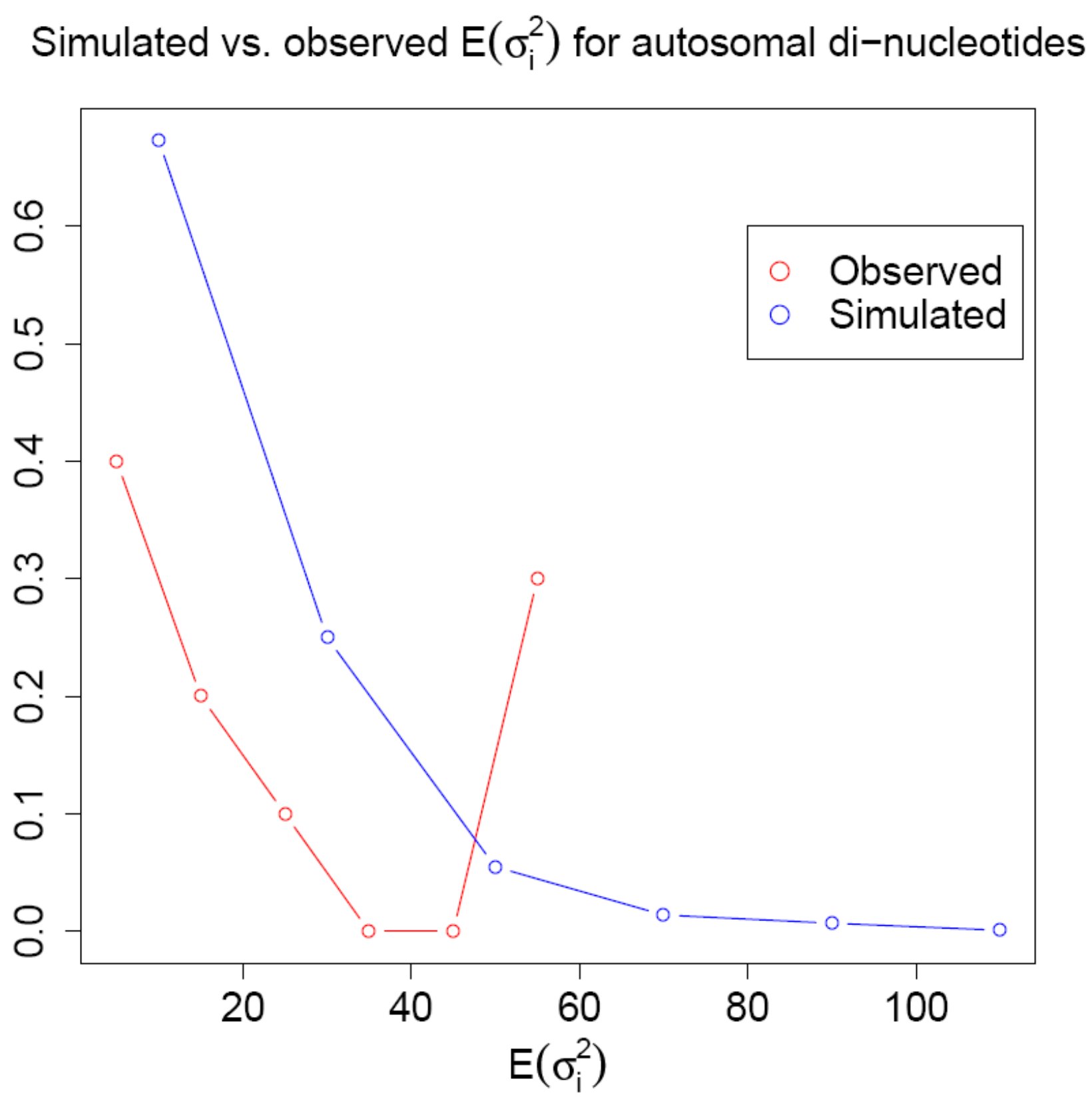

Simulated vs. observed $E(\sigma_i^2)$ for autosomal di−nucleotides
Observed
Simulated
0.0
0.1
0.2
0.3
0.4
0.5
0.6
20
40
60
80
100
$E(\sigma_i^2)$
