# Supplementary material for: Genetic Structure of Chimpanzee Populations
Source: PLoS Genet. 2007 Apr 20;3(4):e66. doi: 10.1371/journal.pgen.0030066 (PMC1853122; doi:10.1371/journal.pgen.0030066)
Supplement: Supplementary file 4 [file pgen.0030066.st001.pdf]

Name	location on chromosome	Locus or alias	Rep	Physical Map	Informativness	# alleles	allele size range	
GATA32C10	Y	DYS391	4	13112029	1.07515	5	289	317
GGAA4B09N	3	D3S2403	4	13147403	1.01563	17	204	269
GATA104	7		4	143115408	0.997608	27	173	227
GATA129H04	1	D1S3721	4	41139784	0.965041	29	188	255
GATA164B08P	3	D3S4545	4	8500000	0.901269	27	213	258
GATA11A06	18	D18S542	4	11482759	0.898777	30	167	210
GATA61E03	6	D6S1051	4	36679852	0.894083	12	207	251
ATTT030	6		4	9540286	0.881354	12	104	136
GATA176C01	2	D2S2972	4	102193472	0.864309	29	198	270
GATA71H05	16	D16S769	4	26125312	0.863574	25	242	300
ATA27A06P	12	D12S1042	3	28000000	0.862575	10	116	146
GATA43A04	1	D1S1653	4	155149568	0.856855	29	116	229
GATA14E09	8	D8S2324	4	74304288	0.854694	13	184	218
GATA116B01N	2	D2S2952	4	8099777	0.852129	20	142	207
GATA50G06	15	D15S643	4	57429880	0.842507	19	187	259
ATA25F04	Y	DYS392	3	21478942	0.840013	7	244	265
UT7544	19	D19S559	4	50022064	0.839055	17	148	177
GATA43C11	7	D7S1804	4	131695864	0.836512	20	214	298
GGAA21G11L	14	D14S617	4	90192832	0.835032	15	131	187
GGAA3A07M	1	D1S1612	4	7827531	0.810623	26	123	185
GATA29A01	6	D6S1959	4	20020072	0.80535	9	146	178
GATA28F03	4	D4S3248	4	60024876	0.795523	13	239	267
TCTA017M	9		4	116500000	0.795131	18	158	200
AGAT120	22	SNP343411	4	19665174	0.791061	22	251	293
GATA25A04	17	D17S1299	4	39367576	0.790268	19	172	218
GATA69C12	X	DXS6810	4	41949628	0.772492	17	195	234
GATA91H06M	12	D12S1301	4	42348912	0.765451	17	87	129
GATA7F05	3	D3S3039	4	73763232	0.765164	9	280	312
GATA129D11N	21	D21S2052	4	27740436	0.759255	6	109	129
GATA8C04	17	D17S974	4	10719277	0.756707	10	185	217
ATA11D10M	6	D6S1021	3	104719488	0.744306	14	127	150
GATA68A03	5	D5S2501	4	110112528	0.74394	21	278	386
UT5029	12	D12S297	4	50899320	0.738263	12	201	245
GATA23D03N	2	D2S1363	4	227232144	0.733393	10	180	220
GATA24F03	7	D7S1819-D7S3056	4	4237377	0.731333	17	146	188
GATA4H09	1	D1S549	4	216700144	0.725471	13	149	205
GATA29F06z	20	D20S477	4	22407060	0.718056	15	225	260
GGAA9D03	17	D17S1294	4	28527738	0.716046	11	216	256
GATA74A05	14		4	32823604	0.713761	25	234	310
GATA50F11	1	D1S1609	4	241012608	0.711939	15	136	180

AAT243	1		3	232893344	0.707789	12	162	198
GATA84E11	5	D5S2505	4	5869851	0.707569	15	249	309
GATA81B01	6	D6S1277	4	164207024	0.705836	9	262	302
GATA45B10N	20	D20S480	4	52542632	0.705186	14	260	310
GATA42A03	20	D20S478	4	37917456	0.700475	17	235	303
ATA19H08	14	D14S592	3	59386896	0.698231	12	204	237
GATA49C09N	17	D17S1290	4	56806088	0.694608	17	169	205
ATA23G05	18	D18S858	3	53046080	0.687458	9	178	202
GATA64H04	18	D18S877	4	24977176	0.683377	12	117	145
GATA72E05M	X	DXS7132	4	63522128	0.681433	17	252	299
GATA4D07	2	D2S1334	4	136667216	0.676265	14	310	366
GATA187D09N	9		4	10220368	0.670194	9	172	204
GATA6D09	18	D18S851	4	48359408	0.669634	16	248	288
GATA52B03	X	DXS6807	4	4204887	0.669253	8	253	281
SDF1	Xq/Yq	DXYS154	2	45879692	0.666971	14	218	244
AAT262	12		3	3885406	0.66528	9	260	284
TTCA006M	17		4	76200096	0.661042	6	152	185
TTA024	8		3	24328004	0.659971	8	157	178
GATA63D12P	12	D12S1064	4	89325736	0.65937	20	152	210
GATA72E11	20	D20S602	4	7321075	0.657464	12	233	276
GATA7C06M	5	D5S1470	4	32537792	0.65294	20	174	241
GATA169E06	14	D14S1433	4	73603616	0.652587	11	156	196
GATA88F09	10	D10S1435	4	2197272	0.646532	19	272	322
AAT013	5		3	173312832	0.643743	10	253	277
ATA70B03	14		3	21697972	0.638726	9	148	172
GATA24D12P	7	D7S1818	4	48900000	0.631705	12	170	214
AAT261	9		3	90461120	0.628635	11	117	147
GATA63F08P	7	D7S2202	4	138100000	0.626113	14	140	180
GATA63F09	11	D11S2365	4	55981152	0.625502	14	226	258
GATA198B05N	22		4	16025204	0.625136	9	93	125
GATA11C08P	13	D13S1807	4	53100000	0.624227	10	167	197
GATA119B03	7	D7S3047	4	9219316	0.623323	11	144	180
b001ve9	9		2	134314912	0.622404	10	145	163
GATA157H01	18		4	71	0.622252	14	169	203
GATA7E12	18	D18S1364	4	61549128	0.619609	17	144	187
GATA22F11NZ	3	D3S2427	4	177105712	0.616406	19	155	203
GGAA22G01	13	D13S895	4	107900000	0.614975	3	127	139
GGAA4A12	14	D14S588	4	68210328	0.61258	7	81	121
GATA134B03	5	D5S2845	4	22436148	0.611628	7	139	159
ATA43A10M	17	D17S2193	3	67144912	0.611544	13	86	114
ATA28B11	6	D6S1031	3	77456936	0.605005	12	254	287
AGAT021	2		4	242669056	0.60499	17	198	246

GATA72G11N	2	D2S1780	4	3309619	0.592942	15	300	344
TAA005	9		3	1641037	0.592532	9	140	167
GATA118G10	7	D7S3046	4	67964440	0.592141	25	277	350
ATT198Z	14		3	102317440	0.589519	6	143	167
TCTA023	14		4	30524656	0.586154	9	158	194
GATA72G09N	4	D4S2632	4	35601324	0.584949	14	134	202
GATA165B12P	X		4	115600000	0.582124	7	121	149
GATA8B05M	3	D3S1768	4	34596172	0.581425	8	167	199
GATA11E02N	6	D6S2410	4	50690384	0.581052	18	230	266
GATA30G01	11	D11S2002	4	79691712	0.580124	14	232	272
GATA48D07	9	D9S930	4	110616048	0.57717	16	262	322
GATA62F03M	9	D9S2169-D4S2624	4	5190409	0.576894	15	256	308
GATA178F11z	18		4	2113098	0.57404	12	271	319
035xb9	7	D7S2477-AFMb035xb9	2	2573040	0.573089	17	140	172
GATA133A08Q	1		4	105636168	0.570328	8	98	134
GATA87G01	10	D10S1432	4	74004000	0.568268	12	162	185
GATA73D10L	7	D7S2204	4	77738976	0.562774	16	205	273
282zh9	9		2	14344486	0.55937	14	155	189
GATA31F01P	X	DXS6789	4	94221096	0.559078	10	105	145
ATA27H09	2	D2S1353	3	159761472	0.551058	8	126	150
ATA47D07	1	D1S3720	3	20381404	0.548069	6	115	133
GATA138B05	5		4	71485952	0.547897	8	162	194
GGAA2A03	19	D19S433	4	35108868	0.546597	14	163	215
GATA62A12	4	D4S2623	4	111348528	0.544089	11	177	225
GTTTT001P	15		5	28700000	0.540089	8	190	210
AAT269	20	SNP481439	3	48642016	0.539948	10	226	249
GATA8F07	2	D2S405	4	29451182	0.538375	10	245	273
CAC002	X		3	36511000	0.536974	10	190	211
GATA51D11	5		4	133000000	0.536453	3	102	110
ATA29C07L	1	D1S3462	3	228985520	0.53243	10	237	264
AGAT126	5		4	150833968	0.531829	12	195	215
ATA21A03	10	D10S1221	3	56874496	0.530667	7	195	213
046xf6	20		2	57241284	0.525477	9	119	143
ATA9B04N	11	D11S4459	3	56289880	0.521225	9	234	258
GATA52A04M	2	D2S1384	4	205400000	0.521051	16	130	175
GATA74E02Z	14	D14S742	4	20191104	0.520872	13	336	387
GGAA20G04	2	D2S1399	4	148420880	0.519483	12	125	181
ATA2A03	4	D4S2361	3	85463832	0.518723	7	137	155
Mfd232	19	D19S246	4	55647456	0.517577	12	137	177
GATA4H01	12	D12S395	4	118600416	0.51354	10	201	231
GATA28D01M	11	D11S2000	4	105096456	0.513314	17	196	236
ATAG089	18		4	55706068	0.512244	8	156	176

GGAT1A4	10	D10S2327	4	80056656	0.508474	10	196	232
UT6540	6	D6S942	4	765498	0.500161	10	180	220
b329we5	14		2	20511740	0.495662	12	85	109
GATA8G10M	8	D8S1110	4	53230932	0.494075	20	251	311
AGC001b	6		3	170727440	0.493931	13	177	240
AAC023	3		3	67951712	0.493794	9	204	228
TTTA049	1		4	244823712	0.490819	4	230	246
ATA42G04P	9		2	110300000	0.490451	10	180	207
GATA7D12	9	D9S301	4	69259840	0.48855	13	197	233
ATA50C05	6	D6S2434	3	14054316	0.488046	13	200	242
TATC043	X		4	144373552	0.486312	13	234	286
GATA4E11	2	D2S410	4	116336072	0.48578	11	140	180
GATA26E03M	8	D8S1132	4	107285456	0.481786	15	84	131
ATAA018P	8		4	21257580	0.480963	7	101	125
AGAT113Z	13		4	109987640	0.477992	8	144	172
GATA23E06L	11	D11S1998	4	117235408	0.477661	9	124	156
GATA81D05	Xq/Yq?	DYS395	4	2776258	0.477268	11	98	146
GATA10G07	4	D4S3243	4	81390768	0.474526	8	126	154
ATT015	4		3	13677525	0.474449	11	262	283
GATA6A05	1	D1S551	4	82317888	0.47424	14	170	218
ATA23A10M	5	D5S1480	3	144172224	0.471264	13	207	240
GATA88H02N	15	D15S822	4	24953448	0.470898	19	230	282
GGAA3C07	21	D21S1437	4	20568710	0.467513	9	99	131
b037wf5	5		2	149944112	0.467054	5	143	151
ATA22D02	10	D10S1222	3	128735152	0.466558	5	221	233
GATA24H01N	4	D4S2367	4	68286184	0.466145	11	111	147
AGAT130	5		4	125001592	0.465374	5	212	222
ATAAC002	12		4	10655885	0.464438	9	265	310
ATA20G07M	5	D5S2488	3	180129	0.463163	18	253	281
ATA7D07	18	D18S1357	3	56968832	0.459614	6	117	135
GATA6G12	3	D3S2398	4	190741936	0.456669	11	258	302
GATA3H06M	5	D5S1462	4	96454600	0.452636	12	160	208
GATA145D10N	5	D5S2849	4	3477322	0.450111	9	182	218
GATA49D12N	12	D3S2395	4	8048872	0.444495	15	158	197
GATA31B09	14		4	24645920	0.441614	11	277	339
GATA188F04	21	D21S2055	4	37800000	0.440507	16	85	127
GATA23A02	2		4	236306288	0.439177	14	143	175
ATCC001	10		4	6398267	0.438679	7	300	324
242xb10	5		2	171273168	0.435721	21	148	176
GATA29B01L	19	D19S589	4	58498396	0.435593	11	168	208
GATA2H09	5	D5S816	4	135377728	0.435218	8	225	253
ATA82B02N	18		3	67062488	0.429597	8	169	190

GATA70B08	21	D21S1446	4	46893788	0.42483	17	184	227
GGAA2f11N	10	D10S677	4	95629024	0.424812	11	177	229
UT7136	22	D22S532	4	44399728	0.418487	11	155	195
PAH	12	PAH	4	101785624	0.410725	11	213	261
GATA50D10	8	D8S1108	4	136262704	0.408391	10	244	280
GATA3C02M	3	D3S1744	4	148413424	0.404977	8	128	156
ATA28C05	X	DXS6795	3	22605926	0.397311	2	270	273
ATA77F05	14		3	23739004	0.397112	11	185	206
GATA62A04	5	D5S1505	4	119177864	0.395697	11	187	239
GATA81D12M	16	D16S2624	4	71511312	0.394281	7	117	141
ATA3A07	16	D16S748	3	3370871	0.385995	10	175	202
GATA22G05M	4	D4S2366	4	6549327	0.3859	8	121	149
MFD455	9	AAT052	3	600000	0.383623	13	284	305
GATA175H06M	9		4	8100100	0.37721	8	154	186
ATA24A08	15	D15S652	3	90247136	0.376967	12	273	309
ATA10H11	3	D3S2409	3	49377096	0.376416	8	103	130
GATA7H10	5		4	166808880	0.376099	11	136	175
GATA63A03N	15	D15S659	4	44090064	0.375089	9	174	206
GATA90D07N	11	D11S2371	4	73231440	0.374593	8	173	209
GATA84C01	10	D10S1430	4	12740995	0.369417	8	132	160
GATA13G11	7	D7S817	4	31800000	0.360364	14	157	205
GATA12H10	2	D2S427	4	232408800	0.356479	9	215	245
TTA001	16	MFD466-TTA001	3	78886368	0.354042	10	263	290
GATA4G12	2	D2S434	4	218779040	0.353614	11	270	314
GATA64D04	4		4	171280912	0.348159	11	229	265
ATA7B01	20		3	71424552	0.347888	9	95	119
TATC028	1		4	165578496	0.34683	4	251	258
GATA69E12M	2	D2S1394	4	731133696	0.345717	10	143	179
GATA13	18	D18S535	4	36400776	0.345583	13	119	151
GATA41G07M	7	D7S1802	4	20449620	0.345486	15	140	196
CATA002Z	16		4	23716036	0.344594	4	220	232
SraP	2	(TPO)	4	1500000	0.342851	6	116	136
GATA23F05	7	D7S1799	4	103755536	0.342575	11	147	191
GATA48H04	X	DXS6805	4	115261312	0.339205	11	312	344
ATA27F01	21	D21S1440	3	38062024	0.338961	10	138	165
AAT266	5		3	162159216	0.338139	10	252	273
GATA5E06P	9	D9S304	4	32313900	0.336159	10	138	178
AAAT126	9		4	98769696	0.333435	9	137	173
AGAT060	18		4	6303943	0.331218	10	263	299
GATA67D03	5	D5S2500	4	58713076	0.329758	12	133	181
GATA51B02M	13	D13S796	4	105586968	0.328813	12	132	176
GATA21D04	5	D5S1457	4	41079360	0.327522	10	99	135

GGAA5F09	1	D1S1679	4	160000000	0.320279	12	136	184
ATA5A09N	13		3	28870680	0.319214	10	141	162
ATA31G11P	10	D10S1412	3	9200000	0.318828	10	158	185
ATA1B07	11	D11S1993	3	43574408	0.31746	9	200	227
GATA21F05	9	D9S922	4	78425464	0.317248	11	271	315
GATA189C06M	7	D7S3070	4	150958848	0.317083	10	157	193
GATA48E02	11	D11S1981	4	17050512	0.315969	17	130	174
UT721M	8	D8S373	4	144330496	0.315736	14	170	218
GATA31	6	D6S474	4	112924672	0.310295	9	131	163
GATA22F09P	16	D16S3253	4	54565224	0.299639	10	184	215
ATA79C10	1	D1S3776	3	34055604	0.294441	10	232	250
GATA27C08P	3	D3S2432	4	32136946	0.290464	11	135	168
GATA27G03	4	D4S2368	4	169412176	0.288227	7	300	324
GATA51D03	20	D20S482	4	4501279	0.286317	9	143	183
GATA47F05	20	D20S481	4	44453712	0.286093	11	209	241
GATA11C06N	16	D16S539	4	86167736	0.285508	10	144	170
GATA124C08N	1		4	98148272	0.28374	9	111	147
GGAT3F08	Xq/Yq	DXS9900	4	185625776	0.283391	7	150	174
GATA71C09	10	D10S1425	4	119004744	0.280012	3	144	152
ATA12D05P	6	D6S1022	3	28700000	0.279184	9	116	143
GATA27Z	9	D9S299	4	104070264	0.275011	9	201	233
ATTC013	20		4	9533638	0.273604	9	86	118
ATA26B08	4	D4S2394	3	130756656	0.270905	7	241	259
4PTEL04	4	D4S3360	2	105905	0.270571	8	168	182
GATA11H10	2	D2S1360	4	17476470	0.270055	11	120	148
ATT070	8		3	14933155	0.269669	6	188	203
GATA7F09	12		4	5837039	0.266069	9	258	286
GATA88G05	2	D2S1790	4	85049832	0.26092	9	292	328
GATA6C01	12	D12S373	4	16906264	0.259527	8	192	224
GATA129B03N	20	D20S1143	4	15293073	0.258607	9	160	192
Mfd235	19		4	38789996	0.258443	9	187	215
GATA124E07	X	DXS9896	4	28698586	0.257784	8	171	199
GATA124F08	1	D1S3761	4	206052656	0.252991	9	213	245
GATA11A11P	5	D5S1456	4	169013408	0.250081	13	175	211
GATA32F05	12	D12S2078	4	126314240	0.248127	11	246	274
GATA61G06	7		4	5374993	0.243222	9	90	117
GATA168F06	14	D14S1434	4	93298400	0.241214	7	212	236
GGAA20C10M	8	D8S1477	4	32124836	0.238203	7	132	156
ATG021	12		3	13669000	0.236508	6	95	110
ATA22E01	3	D3S2418	3	193637920	0.23465	3	96	102
GATA7G10	13	D13S317	4	80520064	0.233762	8	155	199
GATA177C03N	18	D18S1371	4	71237728	0.230902	8	117	145

GATA184A08	6		4	147947632	0.22954	12	153	181
b014ye5	9		2	89826616	0.226578	6	176	186
ATA22G07P	6	D6S1027	3	168965744	0.225575	6	118	136
GATA73D11	5		4	114294904	0.224407	8	208	240
ATA6C09P	6	D6S1035	3	159936288	0.223219	10	120	147
AGAT010	3	MFD433-AGAT010	4	2693695	0.222193	5	268	276
GATA85A04M	12	D12S1300	4	97003408	0.220424	9	104	140
ATA42G12M	1		3	106430320	0.213899	9	170	194
GATA158H04	17	g10693	4	6533636	0.213877	8	162	194
ATA18A07M	9	D9S910	3	97003728	0.211294	8	109	127
GATA6B02P	8	D8S592	4	117400000	0.210334	7	141	165
GATA72H07	1	D1S2134	4	47651184	0.204464	10	265	301
GATA131D09	3		4	4288116	0.204275	9	131	159
GATA68F07	3	D3S2460	4	118723280	0.203331	11	147	175
ATA092	5		3	140333584	0.202629	8	211	232
GATA90G11M	14	D14S748	4	46071316	0.199056	8	221	253
AGAT128	3	SNP504344	4	62464944	0.197684	10	242	286
GATA23F06L	11	D11S1999	4	10684295	0.19454	3	88	96
GATA70E01	4		4	20864456	0.192423	8	158	190
GATA66B04	19	D19S714	4	15589133	0.188143	12	224	271
AAT107	16		3	59668096	0.187152	7	194	230
GATA73D01	3	D3S3038	4	21924468	0.185125	9	167	199
GATA23F08	6	D6S1040	4	130966320	0.183442	7	257	289
GATA50C10	5		4	116007288	0.181308	8	298	322
GATA3E10	5	D5S817	4	11638225	0.181035	8	244	272
GATA43H01M	14	D14S608	4	26839482	0.17547	11	193	229
GATA68H04	6	D6S1056	4	94093104	0.174924	11	225	257
GATA22H04M	9		4	77000000	0.173861	8	191	219
GTT028	1		3	166402000	0.170747	7	169	187
GATA144D04	X		4	44059432	0.167258	4	230	242
GATA115E01N	10	D10S2470	4	92029192	0.166025	11	226	270
ATA37D06	22	D22S1045	3	35779336	0.163614	8	137	158
GATA11E09	4	D4S1644	4	142329088	0.163119	9	174	206
GATA64F08	13	D13S800	4	71672696	0.163084	9	283	323
TAGA006	4		4	119476504	0.154063	9	228	260
GATA121A08N	10		4	69857176	0.152802	8	176	208
TATC010	7		4	7477116	0.15153	8	212	240
GGAA23C05N	10	D10S1248	4	130566864	0.148696	9	229	261
GATA41A01	8	D8S1136	4	65300000	0.147956	5	225	241
GATA107	4	D4S1625	4	144078576	0.143052	8	182	210
GATA64G07	9	D9S934	4	116471800	0.141294	8	194	222
UT1772	20	D20S164	4	57738776	0.138713	2	183	187

GATA48B01	1	D1S1660	4	195899600	0.130989	7	222	246
GATA71D01	2	D2S1776	4	169847760	0.126116	5	276	300
AAT245	17	SNP427776	4	55774500	0.124988	3	152	156
GAAT1A4N	8		4	99146360	0.114135	4	128	140
AGAT144	X	SNP554669	4	8120196	0.113157	4	312	328
GATA26G09P	1	D1S1596	4	59100152	0.109177	5	116	132
GATA52A12	5	D5S1501	4	78552528	0.0940846	4	84	90
AGAT125	9	SNP446030	4	127327088	0.0872513	5	159	175
GATA151F03N	15	D15S1507	4	63055828	0.0802105	5	204	220
GATA172D05	X		4	111938560	0.0772652	3	103	109
ATGG002	14		4	97207552	0.0520637	2	204	212
ATA26D07	13	D13S779	3	99201952	0.051547	2	157	159
GTTT002	7	MFD442	4	155405904	0.0482547	3	267	275
AAT071	3		3	151483952	0.0473206	5	159	166
ATA34G06	3	D3S4523	3	122212976	0.0395617	3	228	234
TTA032z	6		3	7637946	0.0279654	2	171	174
ATA24F10	10	D10S1225	3	64099648	0.0135139	2	166	169
ATA29C03	10	D10S1230	3	122407280	0.00337425	2	114	117
